# The interplay of personality and attitudes toward own aging across two decades of later life

**DOI:** 10.1371/journal.pone.0223622

**Published:** 2019-10-09

**Authors:** Anna E. Kornadt, Jelena S. Siebert, Hans-Werner Wahl

**Affiliations:** 1 Department of Psychology, Bielefeld University, Bielefeld, Germany; 2 Department of Psychology, Heidelberg University, Heidelberg, Germany; Aalborg University, DENMARK

## Abstract

Big Five personality traits are assumed to be linked with attitudes toward own aging. Since both constructs have central importance for the aging process, it is surprising that to our knowledge no study so far comprehensively addressed their mutual connection over time. We used data from the ILSE study, a longitudinal study capturing personality and attitudes toward own aging at four measurement occasions, spanning 20 years and including two participant cohorts in midlife (*n* = 501; born 1950–52) and later life (*n* = 500; born 1930–32). Dual latent change score models showed that personality was longitudinally related to change in attitudes toward own aging: Lower Neuroticism, higher Conscientiousness, and higher Openness predicted more positive attitudes, whereas the direction of the effect for Extraversion varied by time. Furthermore, the role of personality seems to be confined to certain sensitive periods in midlife and early old age. Contrary to our expectations, attitudes toward own aging had only marginal longitudinal impact on the Big Five. Our results shed light on the developmental co-dynamics of personality and subjective perceptions of aging across the second half of the lifespan.

## Introduction

Individual views on aging are central psychosocial variables in the aging process–what people think about older people and their own aging influences how they age themselves [1–3]. Views on aging encompass a variety of different constructs, such as age stereotypes (socially shared beliefs about the process of aging and about older people as a group), subjective age (the age people feel like), and self-perceptions of aging (expectations and evaluations of one’s own aging). The latter has also been termed attitudes toward own aging in the literature [4, 3], and in recent years, research on views on aging in general and attitudes toward own aging in particular has flourished. There is abundant evidence that attitudes toward own aging impact developmental processes and outcomes in mid- and later life. For instance, as people get older, those with better attitudes toward aging show less negative affect, better health behaviors, better health and decreased mortality, better cognitive functioning, and lower risk of dementia compared to people with more negative attitudes toward their own aging, e.g. [5–11].

Despite this evidence, what might drive the characteristics and development of attitudes toward aging has been researched to a lesser degree. Variables that seem to influence attitudes toward own aging across adult life are psychological and physiological resources, such as objective and subjective health and well-being [12, 13], or personality factors such as rigidity [14]. Another variable that has received some attention is personality conceptualized as the Big Five personality traits (e.g., [15]). It has been found that personality traits and attitudes toward own aging are related (e.g., [16]), however, studies that include large samples in broad age ranges with several measurement occasions across a considerable observation interval are rare, leaving the developmental dynamics of attitudes toward own aging little understood. Our study thus aimed at investigating the developmental co-dynamics of attitudes toward own aging and personality in mid and later adulthood.

### Traditional argument: Personality influences attitudes toward own aging

Some research on the antecedents of attitudes toward own aging has followed approaches of classical attitudinal research in social psychology. The assumption is that personality, i.e. the Big Five, as a fundamental trait characteristic can be conceived as the basis of attitudinal variables [17]. Consequently, studies show that personality traits predict what people think about old age and their own aging. Most recently, targeting individuals aged 60 and above, Bryant and colleagues [16] found that Neuroticism, Extraversion, and Agreeableness were prospectively related to attitudes toward own aging (measured with the Attitudes to Aging Questionnaire; [18]) 10 years later: People higher in Neuroticism had less positive attitudes toward aging as a time for psychological growth, whereas both Extraversion and Agreeableness were negatively related to the perception of age as a time of social losses.

Besides, lifespan developmental psychology also provides a theoretical rationale for the possible influence of personality traits on attitudes toward own aging. As people move through the lifespan and their own aging process, their attitudes toward aging are assumed to develop and change among others as a function of experiences that are somehow related to age (e.g., [19, 1]). Personality influences the selection of certain situations and environments and consequently, the acquisition of experiences (e.g., [20]). People with specific personality characteristics might thus be predisposed to make (or avoid) certain experiences related to age. For example, older persons high in Openness to Experience might more readily engage in a senior activity program and thus experience less boredom and loneliness in old age, which in turn might impact their attitudes toward aging. Furthermore, personality characteristics also shape the way people interpret experiences and the situations they arise in. For example, people with higher Neuroticism tend to focus on negative aspects of a situation and thus interpret the situation in a more negative light (e.g., [21]).

Several studies that investigated the relationship between personality and views on aging build on this theoretical basis. Cross-sectionally, Moor, Zimprich, Schmitt and Kliegel [22] found that Neuroticism and attitudes toward aging were negatively related. Using longitudinal data from the same study, Miche and colleagues [4] supported this finding within a 12-year time span for participants aged 42–46 at baseline. In another longitudinal study Shenkin, Laidlaw, Allerhand, Mead, Starr, and Deary [23] used a different, multidimensional measure and found that, controlling for a number of other factors, all Big Five traits were longitudinally related to attitudes toward aging as psychosocial loss (Neuroticism positively, all others negatively) and that attitudes toward aging as physical change and psychological growth were predicted by high Extraversion, Agreeableness, Openness and Conscientiousness. The respective relations were strongest for Neuroticism (psychosocial loss), Extraversion and Openness (physical change), as well as Extraversion, Agreeableness, and Conscientiousness (psychological growth). Relatedly, Rupprecht, Dutt, Diehl and Wahl [24] recently found that higher Neuroticism was related to the awareness of more age-related losses, whereas Openness and Conscientiousness were related to the awareness of age-related gains. Taken together, previous research supports the assumption that personality might influence the development and characteristics of views on aging. This direction of influence makes also sense considering the fact that personality in adulthood is assumed to be relatively stable, having developed over decades and providing a coherent and consistent frame of reference across life [25].

### The new plasticity argument: Do attitudes toward own aging also influence personality?

Nevertheless, there is recent evidence for personality plasticity and change even in advanced old age (e.g., [26, 27], hence, personality stability seems to decrease again as people grow older [28]. Considering this plasticity as well as the strong impact of attitudes toward own aging on a variety of developmental outcomes described previously it seems plausible that attitudes toward own aging might also influence personality.

Established lifespan developmental theories support this direction of influence. They assume that people’s expectations and wishes and the ensuing motivational implications turn them into active agents in their own development (e.g., [29]). This assumption of self-directed development has recently also received attention in research on personality development [30]. More concretely, goals and values have been shown to influence the direction in which the Big Five characteristics develop (e.g., [31, 32]); however, this line of research has thus far not been combined with views on aging research. This is an important omission since especially people’s attitudes toward their own aging influence goal selection and behavior and thereby set the pattern for further development (e.g., [33]), which might also affect personality traits. For example, individuals driven by the view that old age is a time of leisure and pleasure might wish to stay socially active in old age and engage in more activities, which in turn might keep up or even increase their levels of extraversion. In contrast, individuals with more negative expectations regarding social life in old age might also act accordingly and reduce their activities and social lives, contributing to the documented decrease in Extraversion in older age (e.g., [27, 34]).

Along these lines, Diehl and colleagues [1] argue that views on aging in general play a central role in self-regulation and self-consistency in older age, which might also have implications for personality development in the realm of the Big Five traits. As an important indicator that views on aging influence self and personality related variables, several studies found that what people think of older persons in general becomes integrated into their self-concept and self-descriptions over time, especially when age-related experiences and changes are still expected [35–37]. Views on aging also influence action and behavior selection in the face of age related changes which might have an impact on personality. For example, in stressful situations in which people may also become aware of actually having grown older, having the mindset that a person becomes calm and even-tempered in older age might lead to matching behavioral responses, e.g., reacting level-headed and considerate (cf. [38]). This might be interpreted as *being* more relaxed than in younger years. When repeatedly faced with similar situations and concurrent behavioral responses, this might result in actual personality change in the direction of more Agreeableness or less Neuroticism (cf. [39]). Such a mechanism is also in the interest of self-consistency, i.e. the maintenance of a coherent self even in the light of developmental changes and losses. Self-consistency is a central prerequisite for the maintenance of well-being and integrity and thus adaptation in older age (e.g., [40]).

### Rationale for the current study

The current study aims at integrating previous research and as a consequence providing a more comprehensive test of the relationship between personality and attitudes toward own aging with a strong developmental focus. To address this research challenge, a longitudinal study is needed that assesses both variables at several time points, preferably in a large sample of middle-aged and older adults, for whom aging processes and therefore attitudes toward own aging are (becoming) relevant and imminent. The Interdisciplinary Longitudinal Study on Adult Development (ILSE, [41, 42]) offers such a design and is thus to our knowledge the only existing study suitable for this endeavor. Furthermore, ILSE provides data from two cohorts of older adults, one born in 1950–52 being 42–46 years of age at baseline, one born in 1930–32 being 60–64 years of age at baseline, thus allowing for comparisons between cohorts.

Two previous analyses have already addressed the relationship between attitudes toward own aging and personality in ILSE [22, 4]. However, one of them [22] was cross-sectional, and Miche et al. [4] could only use three waves and thus 12 years of data collection, and not the four waves that cover a time span of more than 20 years. Such a long time-span is quite unique and has not been available in previous research. Furthermore, none of them has tested the bi-directionality of relations, and even though this does not provide evidence for true causal relationships, investigating bi-directionality provides a better empirical test of both theoretically assumed directions of influence than separate studies testing only one direction. By including all variables at all time points and their respective relations, findings for one direction of influence can be controlled for the respective other, providing more confident estimates. Thus, our study is the first that combines both theoretically plausible causal argumentations and empirically tests the directions of influence in one study.

### Expectations regarding the relation of the Big Five and attitudes toward own aging

Considering theoretical models and previous research relating personality and attitudes toward own aging, we expect that personality is longitudinally related to (the development of) attitudes across rather long time intervals such as two decades. Furthermore, considering the well-documented impact of attitudes toward aging for developmental outcomes and developmental regulation, as well as theories about self-directed personality development, we also hypothesize that the relationship between attitudes toward own aging and the Big Five traits is bidirectional. However, taking into account that the Big Five personality traits manifest, stabilize and develop in a decade-long ecology and person-environment transactions [43], whereas attitudes toward own aging are a sub-facet of identity with increasing self-relevance only in later life [2], we assume the influence of personality on such attitudes to be stronger than the other way around.

We refrain from making specific hypotheses for all five traits, since previous studies delivered mixed results. Even though previous findings suggest strongest linkages for Extraversion (with higher Extraversion predicting better attitudes toward own aging) and Neuroticism (higher Neuroticism predicting worse attitudes toward own aging), studies could not consistently confirm these predictions; and there were results for other traits as well. In addition, drawing from assumptions of lifespan theories of developmental regulation and also the pervasiveness and comprehensiveness shown for the Big Five approach in personality research [15], all Big Five traits can in principle be relevant for attitudes toward own aging (and vice versa).

Regarding age differences in the assumed relationships, they may unfold across the whole age range covered in our study. The youngest participants of our sample were around 44 years at the first measurement occasion (see below) and thus well into midlife. On the one hand, individual development in midlife is still strongly determined by normative roles, such as parenthood and employment. Furthermore, attitudes toward own aging might not be relevant yet due to self-identification as a middle-aged rather than an older adult. Thus, the relationship between personality and attitudes toward own aging might be rather weak in this age group. On the other hand, later midlife and early old age seem to be phases of considerable heterogeneity in attitudes toward own aging [4] and first age-related experiences might already take place and have been mastered [4, 36, 44], suggesting that they may represent particularly sensitive periods to study the developmental impact and antecedents of attitudes. In older age, a variety of personal age-related experiences have already been experienced and mastered, this might drive change sensitivity in both attitudes toward own aging and personality [33]. Furthermore, in older age, attitudes toward own aging seem to change more strongly into the negative direction, and this negative change also becomes more normative, which leads to reduced interindividual variability in intraindividual change [4]. Investigating the bidirectional relationship of personality and attitudes toward own aging is thus a relevant empirical question throughout the second half of life.

## Method

### Sample and procedure

We analyzed data from the ILSE study, a population-based longitudinal study in Germany that encompasses four measurement occasions over a time span of 20 years. In 1993/1994 (T1) participants were randomly recruited via city registries in two metropolitan areas in East and West Germany (Leipzig and Heidelberg), which comprise data on all inhabitants of the respective communities. The sample was stratified by birth cohort, gender, and region and consists of two cohorts of adults. The midlife cohort (C50) was born in 1950–1952 (*N*_T1_ = 501) and the later life cohort (C30) was born in 1930–32 (*N*_T1_ = 500). Follow-up analyses were conducted four (1997/1998, T2), 12 (2005/2006, T3), and 20 (2014/2015, T4) years after the first measurement occasion. At each wave, participants were contacted and re-invited to take part in the study via mail and telephone. Data were collected in individual face-to-face sessions by trained interviewers and geriatricians either at the research sites or at participants’ place of residence (i.e., private household or institution).

The midlife cohort was followed from their early 40s to their early 60s, whereas the later life cohort was followed from their early 60s to their early 80s. Sample age and sample size at all time points is presented in Table 1. Of the later life cohort, 10% did not participate at T2, another 30% at T3, and again 51% at T4 (see Table 1). Reasons for dropout were death of participant (53%), severe health problems (12%), lack of interest or time (12%), relocation (13%) and other reasons (10%). Compared to dropouts, those participating in the fourth measurement occasion had better objective (d = .52) and subjective health (d = .23), were better educated (d = .19), showed better cognitive performance (d = .37), had more positive attitudes toward own aging at baseline (d = .37), were more open (d = .18), and less neurotic (d = -.20), but did not differ with respect to other personality traits or gender composition. In the midlife cohort, 10% of the participants did not participate at T2, another 26% at T3, and 11% at T4 (see Table 1). Main reasons for dropout were relocation (38%) and lack of time or interest (22%). 16% of the initial sample died in the course of the study, 7% indicated severe health problems, and 17% dropped out due to other reasons. In this midlife cohort, participants who remained in the sample up to the fourth measurement occasion were better educated (d = .28), had better objective (d = .23) and subjective health (d = .16), showed better cognitive performances (d = .54) and reported more openness to experience at baseline (d = .28), whereas we found no differences with regard to the other four personality domains, attitudes toward own aging or gender. More information on the ILSE study and the sample in general can be found in Sattler et al. [41], Wettstein, Tauber, Kuzma, and Wahl [45], and Miche et al. [4]. The ILSE study was approved by the ethics committee of the Medical Faculty of the University of Heidelberg. After complete description of the study to the subjects, written informed consent was obtained.

**Table 1:** Sample age and sample size at all time points.

Variable	Mid-Life C50			Late-Life C30		
	*M*	*SD*	*n*	*M*	*SD*	*n*
Age T1	43.7	0.93	501	62.5	0.96	500
Age T2	47.6	0.91	446	66.4	0.97	448
Age T3	55.0	0.96	331	73.9	0.91	316
Age T4	63.5	1.18	293	82.8	1.16	152

### Measures

#### Attitudes toward own aging

Attitudes toward own aging were assessed at all four time points with the Attitude Toward Own Aging subscale of the Philadelphia Geriatric Center Morale Scale (ATOA, [46]; see also [4]). The ATOA scale is an established and widely used measure of age-related self-perceptions. In the current study, we used the German translation that has been used and proven in a variety of studies with German samples (e.g., [47, 48, 49]). Participants answered to five statements (e.g. I have as much pep as I had last year) with “*yes*” or “*no*” and latent factors were computed for each measurement point. Internal consistencies for both cohorts were α = .68/.66/.68/.67 (C30) and α = .61/.68/.71/.69 (C50) across measurement occasions.

#### Personality

Big Five personality traits were assessed at all four time points with the German 60-item Neuroticism-Extraversion-Openness Five-Factor Inventory (NEO-FFI; [50]). The NEO-FFI assesses Neuroticism, Extraversion, Openness to Experience, Agreeableness and Conscientiousness on a five-point scale ranging from “*does not apply at all*” to “*completely applies*”, with twelve items each. Mostly, internal consistencies were of acceptable magnitude. Cronbach’s α at all four measurement occasions were: Neuroticism α = .80/.84/.86/.83 (C50) and α = .77/.80/.80/.79 (C30); Extraversion α = .71/.72/.77/.73 (C50) and α = .70/.70/.74/.67 (C30); Openness α = .60/.66/.57/.69 (C50) and α = .46/.47/.53/.55 (C30); Agreeableness α = .63/.69/.70/.67 (C50) and α = .62/.63/.73/.66 (C30); Conscientiousness α = .77/.79/.71/.81 (C50) and α = .73/.76/.81/.78 (C30).

#### Control variables

All analyses were controlled for sociodemographic variables, i.e. gender (male = 1, female = 2), education (number of years at school and university), and–given that health and cognition are both meaningfully related to personality [51, 52] and ATOA (e.g., [12, 10])–also for baseline objective and subjective health status and cognitive abilities at T1. We restricted the inclusion of covariates to those variables assessed at T1 due to reasons of parsimony and the inconsistent measurement of covariates at different assessment waves. Objective physical health was assessed via a medical in-depth examination consisting of an anamnesis (e.g., medical history of the participant), a medical check-up (e.g., sensory function, blood pressure), blood test results, and a geriatric assessment. Geriatricians aggregated the information into an overall physical health score ranging from 1 (*very good*) to 6 (*very bad*). The 6 scores contained clear descriptions (e.g., score 5 for a poor health status, if suffering from a very serious medical condition, which is not immediately life-threatening or if independent living was no longer possible) to allow objective assignment, to ensure clinical significance of the assigned health score and to guarantee reliability. Furthermore, participants rated their subjective health status on a scale ranging from 1 (*very good*) to 6 (*very bad*). Baseline cognitive ability was measured by a composite indicator of four well-established and wildly used subtests of the German version of the Wechsler Adult Intelligence Scale-Revised [53], i.e. information and similarities as indicators of crystallized functioning and block design and picture completion assessing fluid components of cognitive abilities.

### Analyses

**Fig 1:**
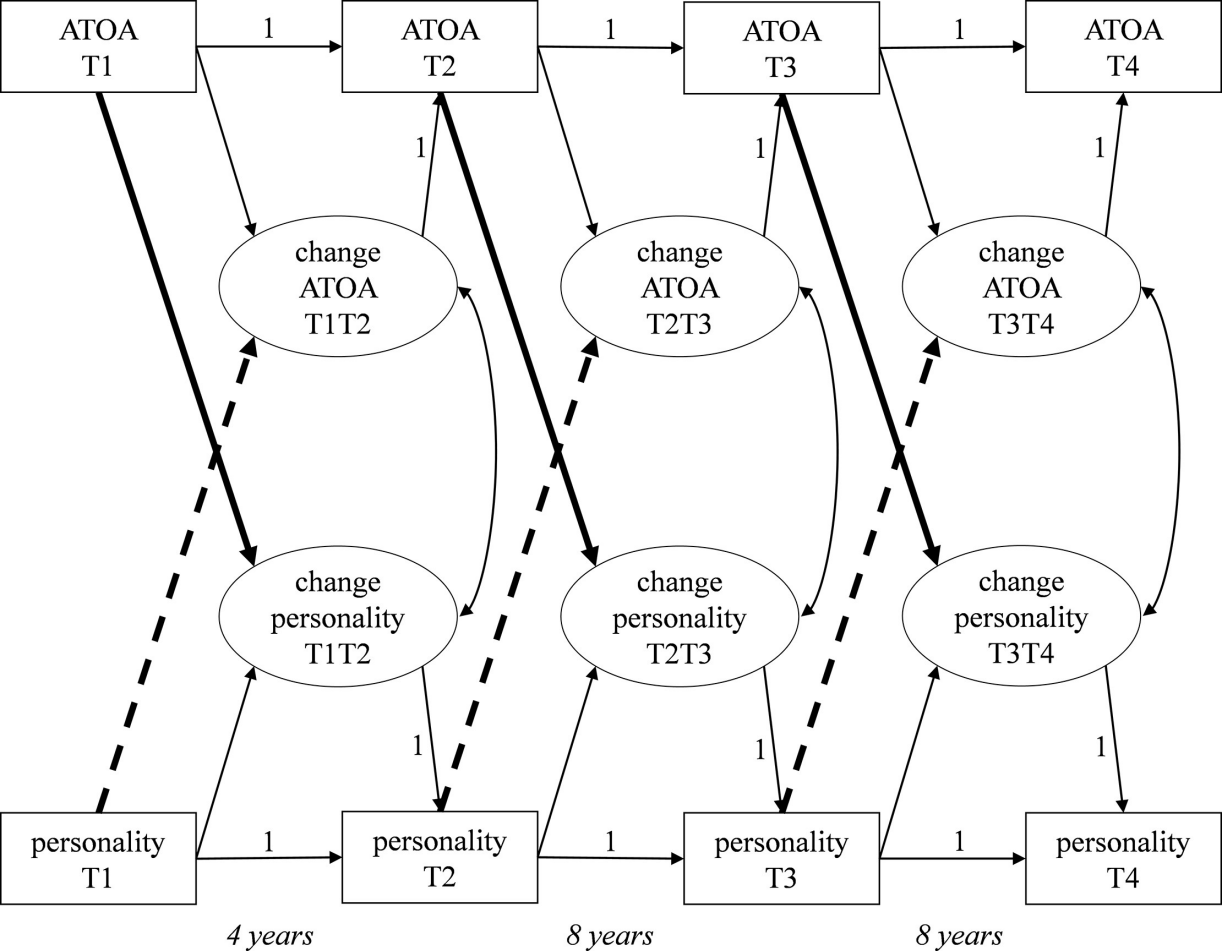
Latent dual change score model linking ATOA and the Big Five over time with level-change-predictions from ATOA to subsequent change in personality (bold lines) and from personality to subsequent change in ATOA (dotted lines).

To illuminate the bivariate, longitudinal relationships of personality and ATOA over 20 years, we ran dual change score models (Fig 1). In these models, a variable at a certain time point (e.g., *T*_*2*_) is a function of this variable at an earlier time point (e.g., *T*_*1*_) and a latent change variable that represents the change from the initial time point to the later time point (*T*_*2*_ *–T*_*1*_)_._ Present models were specified as neighbor change models considering latent change between adjacent time points, namely, change between the first and second, the second and third, and the third and fourth waves, respectively (e.g., [54, 55]). Whereas change was modelled latently, indicators for personality and ATOA were treated as manifest variables. Strong measurement invariance over time was tested in a series of nested models and given for both personality and ATOA in both age groups.

Each model thus consisted of six latent change score variables: Three variables indicating changes in ATOA and three variables indicating changes in personality. Separate models for each cohort (C50 and C30, respectively) and personality factor were conducted resulting in 10 models overall. Notably, we modified level-change covariations (e.g., the covariation between initial level of personality at *T*_*1*_ and T_*2*_ *–T*_*1*_ change in ATOA) as directed path coefficients (i.e. initial level of personality as predictor of subsequent change in ATOA and vice versa). Moreover, change scores of personality and ATOA were allowed to co-vary.

All analyses were conducted with Mplus 7 [56] and parameters were estimated via maximum likelihood estimation available in Mplus. For the evaluation of goodness of fit in our models, we relied on established recommendations [57]. Cutoff values higher than .90 for the Comparative Fit Index (CFI), less than .08 for the Root Mean Squared Error of Approximation (RMSEA) and the Standardized Root Mean Square Residual Index (SRMR) were used to indicate acceptable goodness-of-fit (e.g., [58, 59]). To account for issues regarding multiple testing, the alpha level was set to *p* ≤ .01.

## Results

Descriptive statistics and bivariate associations for all study variables at all measurement occasions are depicted in Table 2 and S1 Table and the personality factors, especially for Extraversion and Neuroticism. In both cohorts and at all time points, Extraversion was positively and Neuroticism negatively related to ATOA. In addition, there were small-sized correlations for Agreeableness, Openness and Conscientiousness in both cohorts, and also some non-significant correlations especially for Openness in the C30 and for Agreeableness in the C50. In general, bivariate correlations in both cohorts became stronger over time (with some exceptions, especially for Openness), suggesting an increasing connection of both variables with advancing age. Moreover, personality and ATOA were both substantially correlated with sociodemographic and health variables at baseline in both the midlife and later life sample.

**Table 2:** Descriptive statistics for all study variables in the Mid-life (C50) and Late-Life Cohort (C30) at four times of measurement.

Variable	Mid-Life C50			Late-Life C30		
	*M*	*SD*	Range	*M*	*SD*	Range
ATOA T1	1.71	0.30	1–2	1.75	0.27	1–2
ATOA T2	1.71	0.29	1–2	1.75	0.28	1–2
ATOA T3	1.59	0.32	1–2	1.65	0.31	1–2
ATOA T4	1.50	0.32	1–2	1.70	0.30	1–2
Neuroticism T1	1.56	0.58	0.08–3.33	1.48	0.59	0.25–3.42
Neuroticism T2	1.51	0.57	0.08–3.08	1.35	0.60	0–3.08
Neuroticism T3	1.46	0.56	0.17–2.83	1.42	0.62	0.08–3.42
Neuroticism T4	1.50	0.55	0.08–3.08	1.27	0.56	0–3.25
Extraversion T1	2.21	0.47	0.50–3.67	2.38	0.48	1–3.64
Extraversion T2	2.19	0.45	0.58–3.58	2.35	0.46	1–3.75
Extraversion T3	2.13	0.49	0.33–3.67	2.31	0.48	0.75–3.58
Extraversion T4	2.04	0.44	0.83–3.17	2.27	0.47	0.60–3.25
Openness T1	2.16	0.39	1.17–3.67	2.28	0.44	1–3.58
Opennes T2	2.15	0.37	0.92–3.42	2.26	0.46	0.83–3.50
Opennes T3	2.16	0.39	1–3.17	2.30	0.48	0.58–5.75
Opennes T4	2.13	0.40	0.67–3.25	2.32	0.46	1–3.67
Agreeableness T1	2.70	0.39	1.58–3.75	2.63	0.41	1–3.75
Agreeableness T2	2.68	0.38	1.45–3.67	2.64	0.42	1.17–3.92
Agreeableness T3	2.74	0.39	1.58–3.83	2.68	0.40	1.25–3.67
AgreeablenessT4	2.77	0.38	1.58–3.67	2.73	0.40	1.42–3.83
Conscientiousness T1	2.94	0.44	1.45–4	2.93	0.45	0.83–4
Conscientiousness T2	2.93	0.42	1.50–4	2.91	0.45	0.75–4
Conscientiousness T3	2.90	0.46	1.57–4	2.92	0.46	0–3.92
Conscientiousness T4	2.84	0.45	1.83–4	2.96	0.46	0.50–3.92
Education (yrs)	14.07	2.50	8–18	12.89	2.76	8–18
Self-rated health	2.47	0.81	1–6	2.55	0.91	1–6
Objective health	2.27	0.78	1–5	2.47	0.88	1–5
Cognitive abilities	85.10	17.37	7–118	79.05	17.79	16–111
Sex (% female)	48.20	--	--	48	--	--

ATOA = Attitudes toward own aging

All dual change models yielded good to acceptable model fit (Table 3), with all CFI values above the threshold of .91 and all RMSEA scores below .08. As shown in Table 4 ATOA and personality traits revealed a pattern of mean level stability in both cohorts across the 20-year time span with some mean level changes in the first re-test interval. Notably, estimated variances of ATOA and all five personality domains at baseline as well as of all change factors pertaining to ATOA and personality were significantly different from zero (all *p*s < .01), showing that there were substantial between-person differences in initial levels of and changes in ATOA and personality in mid- and later life.

**Table 3:** Model fit indices for the dual latent change score models.

	χ^2^(*df*)	*p*	RMSEA [90% *CI*]	*CFI*	*SRMR*
Mid-life C50					
Neuroticism	92.22 (30)	< .001	.065 [.050 - .080]	.95	.078
Extraversion	78.46 (30)	< .001	.057 [.042 - .073]	.96	.068
Agreeableness	80.03 (30)	< .001	.058 [.043 - .074]	.96	.067
Openness	91.81 (30)	< .001	.065 [.050 - .080]	.95	.068
Conscientiousness	78.59 (30)	< .001	.057 [.042 - .073]	.96	.068
Late Life C30					
Neuroticism	74.13 (30)	< .001	.056 [.040, .072]	.97	.074
Extraversion	81.59 (30)	< .001	.060 [.045, .076]	.96	.077
Agreeableness	115.66 (30)	< .001	.078 [.063, .093]	.91	.080
Openness	107.55 (30)	< .001	.074 [.059, .089]	.92	.073
Conscientiousness	78.81 (30)	< .001	.059 [.043, .075]	.95	.076

C50 = Mid-life cohort; C30 = Late-life cohort; RMSEA = Root mean square error of approximation; CI = Confidence interval; CFI = Comparative fit index; SRMR = Standardized root mean square residual.

**Table 4:** Estimated mean level changes in Attitude Toward Own Aging (ATOA) and personality in the mid-life (C50) and later-life cohort (C30).

	Initial Level T1	Change T1-T2	Change T2-T3	Change T3-T4
ATOA				
C50 Mean (SE) Variance (SE)	1.75* (.01) .07* (.00)	-.76* (.09) .06* (.00)	-.27 (.20) .08* (.01)	-.27 (.27) .10* (.02)
C30 Mean (SE) Variance (SE)	1.71* (.01) .08* (.01)	-.83* (.08) .06* (.00)	-.03 (.23) .10* (.01)	-.45 (.29) .07* (.01)
Neuroticism				
C50 Mean (SE) Variance (SE)	1.48* (.03) .28*(.02)	-.61* (.18) .19* (.01)	-.32 (.54) .27* (.03)	1.33 (.87) .24* (.06)
C30 Mean (SE) Variance (SE)	1.56* (.03) .27* (.02)	-.73* (.16) .14* (.01)	-.08 (.56) .19* (.02)	-.10 (.65) .14* (.02)
Extraversion				
C50 Mean (SE) Variance (SE)	2.38* (.02) .20* (.01)	-.48* (.12) .10* (.01)	-.24 (.25) .13* (.01)	-.65 (.32) .14* (.03)
C30 Mean (SE) Variance (SE)	2.21* (.02) .22* (.01)	-.48* (.10) .09* (.01)	.17 (.25) .12* (.01)	-.81* (.32) .10* (.02)
Agreeableness				
C50 Mean (SE) Variance (SE)	2.63* (.02) .15* (.01)	-.62* (.12) .09* (.01)	-.41 (.23) .09* (.01)	-.52 (.32) .08* (.01)
C30 Mean (SE) Variance (SE)	2.70* (.02) .15* (.01)	-.76* (.12) .08* (.01)	-.17 (.26) .11* (.01)	-.01 (.40) .12* (.03)
Openness				
C50 Mean (SE) Variance (SE)	2.28* (.02) .16* (.01)	-.51* (.11) .09* (.01)	-.38 (.26) .13* (.01)	-.02 (.37) .16* (.02)
C30 Mean (SE) Variance (SE)	2.16* (.02) .13* (.01)	-.95* (.10) .09* (.01)	.52 (.30) .12* (.01)	.-.59 (.29) .09* (.02)
Conscientiousness				
C50 Mean (SE) Variance (SE)	2.93* (.02) .20* (.01)	-.86* (.14) .10* (.01)	-.21 (.32) .13* (.01)	-.39 (.31) .11* (.01)
C30 Mean (SE) Variance (SE)	2.94* (.02) .18* (.01)	-.083* (.13) .09* (.01)	.02 (.28) .13* (.01)	-.02 (.38) .10* (.02)

Standard errors of means and residual variances in parentheses.

* *p* ≤ .01.

### Dual change score model: Associations between personality and ATOA

Results of dual change score models for both cohorts and all five personality traits are presented in Table 5. Correlations between concurrent latent change scores (Table 4) showed significant associations of change in ATOA with change in Neuroticism and change in Extraversion across cohorts, while no correlated change emerged for Agreeableness. Change in Conscientiousness was related to change in ATOA only in midlife, whereas concurrent change with Openness emerged only in old age.

**Table 5:** Model parameters (estimates and standard errors) from the Dual change score Models linking ATOA and the Big Five.

	Level–Change Associations						Change Correlations		
	Level P → Change ATOA			Level ATOA → Change P			Change P ←→ Change ATOA		
	βP1-AT12	βP2-AT23	βP3-AT343	βAT1-P12	βAT2-P23	βAT3-P34	*r*ATP_12_	*r*ATP_23_	*r*ATP_34_
Mid-life C50									
Neuroticism	**-.11** (.05)	-.19 (.10)	.22 (.17)	-.04 (.05)	-.07 (.14)	.54 (.27)	**-.38** (.04)	**-.46** (.06)	.03 (.17)
Extraversion	**.12** (.04)	.05 (.08)	**-.35** (.13)	.10 (.05)	.01 (.13)	-.33 (.22)	**.27** (.04)	**.25** (.07)	-.06 (.14)
Agreeableness	.09 (.04)	-.16 (.08)	.14 (.09)	.04 (.05)	.05 (.11)	-.17 (.17)	.09 (.05)	.05 (.07)	.06 (.10)
Openness	.06 (.04)	-.08 (.07)	.03 (.09)	.03 (.05)	-.07 (.12)	-.07 (.18)	.11 (.05)	.00 (.07)	-.08 (.11)
Conscientiousness	-.04 (.04)	**.21** (.07)	-.09 (.10)	.03 (.04)	-.08 (.11)	-.07 (.17)	**.21** (.05)	**.22** (.07)	.11 (.11)
Late-life C30									
Neuroticism	**-.20** (.04)	.09 (.17)	-.25 (.14)	-.11 (.05)	.03 (.17)	.07 (.27)	**-.18** (.05)	-.26 (.10)	**-.36** (.09**)**
Extraversion	**.12** (.04)	.08 (.09)	-.06 (.14)	.10 (.04)	.07 (.14)	-.23 (.25)	**.15** (.05)	**.28** (.08)	.17 (.14)
Agreeableness	.09 (.04)	-.09 (.10)	.22 (.13)	**.14** (.04)	-.04 (.14)	.46 (.22)	.05 (.05)	.05 (.09)	.17 (.13)
Openness	.03 (.04)	**.31** (.09)	.04 (.16)	-.02 (.04)	.20 (.13)	-.22 (.24)	.04 (.05)	**.30** (.08)	.01 (.13)
Conscientiousness	**.14** (.04)	-.07 (.09)	.10 (.13)	.06 (.04)	.03 (.13)	.00 (.23)	.10 (.05)	.14 (.09)	.20 (.11)

C50 = Middle-aged cohort; C30 = Older cohort; AT = Attitudes toward own aging; P = Personality; 1–4 = Measurement occasions. Standardized estimates are reported with standard errors in parentheses. Analyses are controlled for gender, education, cognitive abilities as well as subjective and objective health at baseline. Values in bold print are significant at *p* ≤ .01.

With regard to our main hypotheses, we found the expected significant effects of personality on ATOA over 20 years, controlling for relevant sociodemographic variables as well as baseline health and cognitive abilities. As depicted in Table 5, all five personality factors were longitudinally related with changes in ATOA, however, these effects varied by participant age and personality domain.

Lower levels of Neuroticism and higher levels of Conscientiousness showed less subsequent decline in ATOA. That is, we found significant predictive effects from Neuroticism to change in ATOA between T1 and T2 in midlife (β_C50T1T2_ = -.11, *p* = .01) and later life (β_C30T1T2_ = -.20, *p* < .001). Conscientiousness was related to change in ATOA in the midlife sample (second re-test interval; β_C50T2T3_ = .21, *p* = .006) and late-life sample (first re-test interval; β_C30T1T2_ = .14, *p* < .001). Moreover, higher Extraversion predicted less subsequent decline in ATOA in both birth cohorts during the first re-test interval (β_C50T1T2_ = .14, *p* = .004; β_C30T1T2_ = .12, *p* = .004). In the midlife sample, however, Extraversion was also negatively related to ATOA (β_C50T3T4_ = -.35, *p* = .008); participants with higher Extraversion at T3 showed more decline in ATOA within the next 8 years. In the late life cohort, Openness at T2 substantially predicted change in ATOA within the next 8 years (β_C30T2T3_ = .31, *p* = .001). Contrary to expectations there was little evidence for a bidirectional effect of ATOA also predicting change in personality. That is, we only found one significant coefficient from baseline ATOA to change in Agreeableness between T1 and T2 in the older cohort (β_C30T1T2_ = .14, *p* = .001).

With regard to the covariates, sociodemographics were partially associated with baseline personality domains, but unrelated to baseline ATOA in both cohorts. Subjective health and in the later-life sample also cognitive abilities and objective health significantly predicted baseline ATOA and to some extent also personality factors.

## Discussion

Building on different theoretical and empirical lines of research that link the Big Five personality factors with attitudes toward one’s own aging, we were interested in the long-term, potentially bidirectional relationship of the two constructs in the second half of life. We used data from the ILSE study which so far followed two cohorts of participants in mid and later life over 20 years, providing a unique, longitudinal sample that covers, taking both cohorts together, 40 years of the human lifespan. Using dual latent change score modelling we found evidence for the relationship between personality and the ATOA scale, albeit mainly in the direction that personality was related to subsequent change in ATOA. Thus, our results support the assumption of the Big Five as important predictors of the developmental trajectories in attitudes toward own aging in mid- and later life. Moreover, we found that the link between personality and ATOA was not universal and consistent across time, but seems to be confined to certain personality factors and developmental periods across adulthood. Seen in general terms, this highlights the need of a lifespan approach when investigating the determinants and consequences of attitudes toward own aging and personality development in later life.

### Traditional view still valid with some qualifications: Personality influences attitudes toward own aging–but not universally

As expected and in line with previous research (e.g., [16, 23]), we found that Neuroticism was negatively related to subsequent change in attitudes for participants in their mid-forties and mid-sixties. These age ranges are interesting, since they represent life phases when age-related changes might be especially salient. The mid-forties might be especially prone to age-related changes in family dynamics and bodily changes (e.g. empty nest, aging parents, menopause), whereas the mid-sixties (in Germany) are strongly characterized by the retirement transition and eventually also by first perceptions of bodily decline. These developmental markers might have a special relevance for the development of attitudes toward own aging while at the same time making them more vulnerable to the impact of Neuroticism due to their stress-eliciting nature. It is interesting that we found no influence of personality on ATOA in latest life, even though both constructs show developmental dynamic at the end of life [27, 4]. Further research needs to address factors and events that are responsible for the orchestration (and lack thereof) of both constructs in different life stages.

Interestingly, we found effects in opposite directions depending on participant age for Extraversion. Whereas for the younger and older cohorts, positive effects emerged between the first and second measurement occasions (in the mid-forties and mid-sixties, respectively), Extraversion revealed a negative effect on change in attitudes toward own aging in late midlife (between the late fifties and early sixties). A possible explanation for this change of direction in early old age may be that more extraverted people are used to engaging in and enjoy extraverted behaviors (e.g., having lots of people around, being energetic). Shortly before and during the retirement transition, when social networks and activities have to be realigned, they might first react with stronger negative attitudes toward own aging than people who are less extraverted and thus value these behaviors less or are content with a more tranquil lifestyle. After the retirement transition is mastered, higher levels of Extraversion might become a resource for more positive attitudes toward aging once again. Hence, this finding might suggests that the resource potential of Extraversion might vary across life (cf. [60, 27]). However, this finding needs to be replicated and tested in more detail in order to be more confidently interpreted.

The effect of Conscientiousness was in the expected direction, and is also in line with recent findings on awareness of age-related changes [24]: More conscientious people reported less decline in positive attitudes toward own aging within the next years. This might be due to the frequently shown positive effects of Conscientiousness on health and mortality across life (e.g., [61, 62]). However, these effects are not visible in our data in older ages, which might point to the fact that this resource might also dissipate when mortality-related processes and functional limitations become graver and directly influence attitudes toward own aging.

Openness only had one effect, predicting change in attitudes toward own aging between the ages of 66 and 74. During this life phase, when work obligations dissolve and participants might have more opportunities to actively shape the gained free time, Openness might have the ideal developmental window to impact attitudes toward own aging. Those with higher Openness see and seize opportunities for new experiences and re-orientation, resulting in positive attitudes toward own aging, whereas those with lower Openness might experience boredom and withdrawal, and thus develop more negative attitudes [63].

Finally, Agreeableness was the only Big Five factor that did not show any effect on changes in ATOA. It may be that the strong socially oriented outward focus of Agreeableness only has a lose connection with self-referring attitudes such as ATOA and respective change. It could also be that high as well as low Agreeableness may buffer against the intrusiveness of age-stereotypes eventually coming from one’s social environment. Future research should investigate this dissociation which stands in contrast to the general relation of personality and ATOA. However, Agreeableness was the only factor where we found a significant effect of attitudes on change in personality: Participants in their early sixties with higher levels of ATOA showed increases in Agreeableness four years later. Due to its singularity, however, we are careful not to interpret this relation too strongly. The effect is however in agreement with research showing that lower subjective age (feeling younger), a rating somewhat similar to positive ATOA, is linked with increased Agreeableness across a 10-year observational period [64].

Taken together, our nuanced long-term approach that covered a large portion of the adult lifespan extends previous research by showing that virtually all Big Five traits (and not only Neuroticism and Extraversion) are meaningfully related to attitudes toward own aging across the second half of life. This is also in line with previous findings showing that personality traits might at least partly mediate genetic effects in the variability of age stereotypes [65], and thus have to be considered when trying to understand the sources of views on aging in general across life. However, we also note that even though we show longitudinal relationships controlled for several important covariates, what exactly underlies this relationship remains unclear.

At the same time, our findings suggest that the strength and direction of relationships depends on developmental timelines, if not developmental deadlines [33, 66]. More concrete, our data suggests an accumulation of effects in early old age (between 62 and 66 years), but also earlier in midlife. Interestingly, these time periods might also be seen as being particularly sensitive for the developmental dynamics of attitudes toward own aging. During these times, the explicit experiences of age-related changes start to accumulate; for example, midlife typically comes with first signs of physical and cognitive aging and early old age is linked with the developmental deadline of the retirement transition, which is culturally framed with the beginning of “being old.” It might thus be the case that personality traits can be used as resources for attitudes toward own aging, as long as certain developmental windows are open and provide leeway for change in different directions (e.g., [66]. Obviously, replication in independent samples is necessary to further strengthen such reasoning.

Besides this interpretation, however, another possible reason for the cohort- and time-specific effects cannot be completely excluded: Since the participants from our two cohorts grew up in different historic times, we cannot disentangle age and cohort effects in our data. This is especially relevant considering that our C30 was born before the war and came of age during and shortly after World War II, whereas our C50 was born in the Fifties and thus grew up during the years of the “Wirtschaftswunder” with increasing prosperity and stability. This also means that both cohorts were subjected to a different discourse with regard to aging during formative years in their development, such as identity development and coming of age, as well as their entry into retirement. For example, the security of pensions or the interpretation of retirement as a time for active engagement vs. enjoying one’s well-earned rest markedly differed by the time both cohorts entered into this transition. This might have affected the size and timing of the relation between participants’ personality (change) and their attitudes toward aging (e.g., the impact of Openness on ATOA). A comprehensive, cohort-sequential study would be needed to address these effects in more detail.

### Predictive power of attitudes toward own aging has its limits: ATOA seem not to influence personality

Contrary to our expectations, and also in some contrast to the well-documented impact of attitudes toward own aging on a variety of developmental outcomes in later life, we find little evidence for effects of attitudes on personality. Even though we had expected the effects of attitudes on personality to be weaker than vice versa, finding just one substantial link (effect of ATOA on subsequent change in Agreeableness in old age) is surprising, and we can only speculate about the underlying dynamics. One reason might be the typically high stability of personality traits across time, resulting in limited variability left that could be predicted by the ATOA scale. However, other longitudinal studies based on ILSE show that variables such as cognitive abilities and health could predict personality change across 12 years [67, 45], hence this may only be part of an explanation. Another possibility is that attitudes toward own aging might be better suited to predict *lower level* personality constructs, also termed personal action constructs (PACs) in the seminal conceptual article by Hooker and McAdams [68]. These constructs for example encompass goals and motivations, which might be on a similar abstraction level as attitudes toward own aging, and thus different from the higher-order, more stable personality traits. Furthermore, the broad research area targeting the role of views on aging relies on quite a variety of different constructs [3] and some might be better suited than attitudes toward own aging to capture differential expectations and evaluations of aging and to predict personality. For instance, several studies have shown the impact of subjective age on personality, which also impacts developmental outcomes (e.g., [64]). Besides, more general age stereotypes and stereotypic expectations of older adults’ social roles and behaviors might drive personality development in the sense of the Neo-Socioanalytical Theory of personality development (e.g., [69]). By behaving and assimilating to these stereotypes and roles, personality traits inherent in the roles (e.g. becoming more agreeable as a grandparent) are reinforced, which leads to long-term personality change. Thus, operationalizing views on aging with these constructs might be promising regarding the detection of reciprocal effects (e.g., [36, 70]). In addition, a domain-specific approach, linking specific expectations and views on aging to specific personality traits matching in content might be more fruitful than using the very broad, general, and unidimensional ATOA scale.

### Limitations and directions for future research

Our study investigated the bidirectional relation between personality and attitudes toward own aging with an unusually long observational interval of 20 years in samples of adults in middle vs. older adulthood. Limitations which also point into directions for future research are as follows: First of all, even though ILSE is remarkable in the sense that it followed up on most of its participants with extensive assessments over 20 years, and retained a strong participation rate of 42% of participants overall and even 63% in the C50, the advanced age of the C30 resulted in a relatively small remaining sample size of only about 20% of participants at T4 in this cohort. Even though this problem is common in longitudinal studies with older participants, cf. [45; 71], the reduction of sample size might have limited statistical power to detect effects in the older cohort, especially at later measurement occasions, even though the samples size should still be sufficient for our analyses (e.g., [72, 73]). Furthermore, dropout was somewhat selective, especially for the late-life cohort. Participants who dropped out were less open and more neurotic, and had less positive attitudes toward aging, resulting in a positive selection bias for the remaining participants. However, since we were interested in variable relations and not mean levels of personality or ATOA, we are positive that this should not have affected our results too strongly [45]. Still, our results need to be interpreted with this limitation in mind. A further consequence of selective dropout is a possible reduction in variability and range restriction on the respective variables. This might lead to the underestimation of longitudinal effects. Another characteristic of the ILSE study is that participants were recruited in two middle-sized to large German cities, and thus the generalizability of our results in terms of the German population is limited. In sum, our results need to be replicated in different samples, preferably with larger, more representative sample sizes and less (selective) drop out.

Some of the personality scales showed rather low reliabilities, limiting the height of possible bivariate relations. Relatedly, all variables in question were assessed via self-report. In order to reduce possible method-related bias and artificial sources of variance, it would be important to include for example peer- or observer-ratings of personality (e.g., [74]). This would also reduce some of the general variance in the Big Five and social desirability effects, which might lead to a general “good traits go together with positive attitudes” effect that might be responsible for some of the shared variance in ATOA and the Big Five.

A limitation of the design is that the time between measurement occasions was not equally spaced: Whereas there were eight years between the second and third as well as the third and fourth measurement occasion, respectively, T1 and T2 were only four years apart, which might impact the comparability of findings between the measurement occasions. In fact, many of the directed level-change effects emerge within the short time interval between T1 and T2. However, we decided to keep all time points in the analyses in order to profit from the long time-span covered quasi-longitudinally by the two cohorts, and also the larger number of participants at the first measurement occasion. Besides, we also find age group differences in the number of significant effects between all time points, and for the correlated changes, most effects even become significant between T2 and T3. We are thus confident that there is no systematic effect of length of observational interval, however, agree that 10 year intervals are a long time which might make the detection of directed effect difficult.

Furthermore, control variables were only included at the first measurement occasion. Since we found a consistent and interesting relation between subjective health, objective health, and cognitive status on the ATOA scale and personality, it might be worthwhile in future studies and analyses to include the former variables as time-varying covariates and also test whether these variables moderate the relationship between the two constructs of interest. Unfortunately, we were forced to refrain from such eventually more appropriate co-variance analysis due to methodological constraints. Another important limitation with regard to methodological constraints is that previous research hinted at problems regarding measurement invariance between the age groups [4], so we refrained from testing the similarity of associations between cohorts.

In future studies, the mechanisms that link the Big Five personality traits to attitudes toward own aging need direct in-depth investigation. Even though we used a longitudinal dataset, we cannot make any inferences about causality. The relationships we found might as well be interpreted in terms of underlying third variables that affect personality and attitudes alike (e.g., certain experiences, genetic factors, etc). Besides, since the participants from our two cohorts grew up in different historic times, we cannot disentangle age and cohort effects in our data.

It might also be worthwhile to take a closer look at the affordances and constraints of developmental transitions and age-related experiences in later life (e.g. early midlife, retirement transition, advanced old age) and examine how individual differences might interact differentially with views on aging across the second half of life. This might add important facets to the understanding of person-environment transactions relevant for “successful” development across the lifespan at large. Relatedly, this might also help in understanding the different developmental timing of effects. During some periods in life when aging becomes relevant and age-related changes or experiences imminent, attitudes toward own aging might be most sensitive to influence [33] and thus, personality traits and their behavioral and psychological consequences might have the strongest impact. Besides, cohort and historical effects need to be taken into account that might have an effect on people growing older during different historic times.

## Conclusion

Personality traits are broad, relatively stable variables that describe individual differences in people across life. Our study shows that these variables influence how people think about their own aging, albeit not universally but time-, age- and trait-specific. This might be a pathway through which personality influences how people age. Conversely, we found almost no influence of attitudes toward own aging on the Big Five traits. However, our study provides evidence for the developmental co-dynamics of the two variables and how their relationship develops over long time periods across the second half of life. Our study thus illuminates an important determinant of attitudes toward own aging, and opens the door for further studies that link different levels of personality, age-related identity, and developmental outcomes across the lifespan.

## Supporting information

S1 Table Bivariate correlations between all study variables at four times of measurement for each cohort. Values below the diagonal show associations for the midlife cohort (C50), and values above the diagonal represent correlations of the late life cohort (C30). N = Neuroticism; E = Extraversion; A = Agreeableness; O = Openness; C = Conscientiousness. Values in bold print are significant at *p* ≤ .05.
(DOCX)
