# Supplementary material for: The interplay of personality and attitudes toward own aging across two decades of later life
Source: PLoS One. 2019 Oct 9;14(10):e0223622. doi: 10.1371/journal.pone.0223622 (PMC6785129; doi:10.1371/journal.pone.0223622)
Supplement: S1 Table — Values below the diagonal show associations for the midlife cohort (C50), and values above the diagonal represent correlations of the late life cohort (C30). N = Neuroticism; E = Extraversion; A = Agreeableness; O = Openness; C = Conscientiousness. Values in bold print are significant at p ≤ .05. (DOCX) [file pone.0223622.s001.docx]

**Supporting Information**

**S1 Table. Bivariate correlations between all study variables at four times of measurement for each cohort***.*

|  | 1. | 2. | 3. | 4. | 5. | 6. | 7. | 8. | 9. | 10. | 11. | 12. | 13. | 14. | 15. | 16. | 17. | 18. | 19. | 20. | 21. | 22. | 23. | 24. | 25. | 26. | 27. | 28. | 29. |
| --- | --- | --- | --- | --- | --- | --- | --- | --- | --- | --- | --- | --- | --- | --- | --- | --- | --- | --- | --- | --- | --- | --- | --- | --- | --- | --- | --- | --- | --- |
| 1. ATOA T1 |  | **.48** | **.40** | **.29** | **-.34** | **-.32** | **-.24** | **-.21** | **.19** | **.19** | **.20** | .14 | **.11** | .06 | .11 | .10 | **.17** | **.19** | **.13** | **.27** | **.09** | **.11** | .06 | .08 | .05 | **-.34** | **-.30** | **.17** | .05 |
| 2. ATOA T2 | **.48** |  | **.42** | **.38** | **-.35** | **-.39** | **-.33** | **-.36** | **.20** | **.26** | **.25** | **.26** | .09 | .09 | .11 | .08 | **.13** | **.17** | .10 | **.17** | **.20** | **.21** | **.12** | .03 | **.13** | **-.35** | **-.31** | **.18** | -.04 |
| 3. ATOA T3 | **.35** | **.45** |  | **.57** | **-.23** | **-.24** | **-.46** | **-.33** | **.19** | **.19** | **.34** | **.35** | **.23** | .10 | **.23** | .16 | .06 | .09 | **.11** | **.17** | .08 | .09 | **.21** | .11 | **.13** | **-.35** | **-.36** | **.24** | -.11 |
| 4. ATOA T4 | **.31** | **.33** | **.48** |  | **-.31** | **-.30** | **-.37** | **-.52** | .13 | .16 | **.28** | **.43** | **.19** | .05 | .10 | .10 | **.23** | **.19** | **.24** | **.27** | .13 | .13 | **.24** | **.31** | .11 | **-.38** | **-.31** | **.17** | -.10 |
| 5. N T1 | **-.40** | **-.29** | **-.32** | **-.21** |  | **.75** | **.64** | **.63** | **-.36** | **-.35** | **-.36** | **-.39** | **-.17** | **-.13** | **-.19** | -.09 | **-.24** | **-.23** | **-.13** | -.13 | **-.32** | **-.33** | **-.25** | **-.33** | **-.28** | **.31** | **.18** | **-.29** | **.27** |
| 6. N T2 | **-.28** | **-.45** | **-.37** | **-.21** | **.66** |  | **.65** | **.62** | **-.33** | **-.41** | **-.38** | **-.35** | **-.13** | **-.14** | **-.22** | **-.17** | **-.20** | **-.35** | **-.18** | **-.19** | **-.27** | **-.42** | **-.29** | **-.32** | **-.26** | **.26** | **.19** | **-.31** | **.20** |
| 7. N T3 | **-.30** | **-.32** | **-.54** | **-.33** | **.62** | **.63** |  | **.73** | **-.3** | **-.36** | **-.45** | **-.49** | **-.21** | **-.16** | **-.26** | -.11 | **-.17** | **-.28** | **-.26** | **-.23** | **-.27** | **-.29** | **-.41** | **-.31** | **-.24** | **.24** | **.19** | **-.37** | **.20** |
| 8. N T4 | **-.14** | **-.25** | **-.38** | **-.44** | **.50** | **.57** | **.67** |  | **-.19** | **-.29** | **-.36** | **-.48** | -.15 | -.09 | -.15 | -.12 | **-.22** | **-.27** | **-.21** | **-.31** | **-.37** | **-.42** | **-.41** | **-.5** | -.13 | **.29** | **.20** | **-.29** | .15 |
| 9. E T1 | **.22** | **.23** | **.22** | .06 | **-.33** | **-.24** | **-.14** | -.11 |  | **.74** | **.69** | **.61** | **.20** | **.14** | **.17** | .11 | **.24** | **.21** | **.19** | .14 | **.24** | **.28** | **.28** | **.31** | .03 | **-.18** | **-.11** | -.05 | -.03 |
| 10. E T2 | **.22** | **.36** | **.32** | **.13** | **-.25** | **-.37** | **-.22** | **-.15** | **.72** |  | **.74** | **.67** | **.17** | **.12** | **.16** | .10 | **.18** | **.19** | **.18** | .14 | **.28** | **.42** | **.35** | **.36** | .04 | **-.16** | **-.14** | -.06 | -.02 |
| 11. E T3 | **.20** | **.27** | **.40** | **.13** | **-.17** | **-.27** | **-.41** | **-.25** | **.62** | **.70** |  | **.74** | **.24** | **.19** | **.22** | .16 | **.24** | **.26** | **.23** | **.20** | **.23** | **.29** | **.39** | **.41** | .03 | **-.15** | **-.14** | .06 | -.02 |
| 12. E T4 | **.13** | **.29** | **.37** | **.29** | **-.26** | **-.32** | **-.33** | **-.44** | **.56** | **.66** | **.71** |  | .10 | .01 | .08 | .09 | **.21** | **.20** | **.24** | **.21** | **.29** | **.32** | **.39** | **.36** | .01 | **-.24** | -.13 | .01 | -.12 |
| 13. O T1 | **.20** | **.17** | .09 | .08 | **-.09** | -.09 | **-.12** | -.11 | **.15** | **.16** | **.14** | **.13** |  | **.60** | **.62** | **.52** | .04 | .06 | .03 | .06 | -.01 | .01 | .05 | .11 | **.32** | **-.09** | -.04 | **.26** | -.03 |
| 14. O T2 | **.17** | **.20** | **.12** | **.13** | -.06 | **-.11** | **-.11** | **-.12** | **.13** | **.22** | **.18** | **.18** | **.74** |  | **.60** | **.64** | -.01 | .01 | .06 | .13 | -.06 | .02 | .09 | .00 | **.28** | -.08 | -.05 | **.28** | .05 |
| 15. O T3 | **.14** | **.16** | **.14** | **.22** | -.03 | -.05 | **-.15** | **-.18** | .08 | **.12** | **.19** | **.18** | **.63** | **.70** |  | **.70** | .00 | .04 | .05 | .11 | -.06 | .02 | .06 | .09 | **.40** | **-.15** | -.01 | **.29** | -.03 |
| 16. O T4 | **.16** | **.19** | **.13** | .09 | -.03 | -.03 | **-.13** | **-.14** | .07 | **.14** | **.20** | **.22** | **.71** | **.74** | **.67** |  | .01 | .05 | .01 | .07 | -.02 | .01 | .00 | -.03 | **.17** | -.11 | -.04 | **.24** | -.07 |
| 17. A T1 | **.11** | **.16** | -.02 | .07 | **-.20** | **-.19** | **-.11** | **-.17** | **.13** | **.09** | .09 | .06 | .04 | .04 | -.02 | -.02 |  | **.66** | **.62** | **.56** | **.24** | **.18** | **.16** | **.21** | .07 | -.02 | -.04 | .01 | **.16** |
| 18. A T2 | **.13** | **.18** | .00 | .03 | **-.16** | **-.3** | **-.16** | **-.23** | .05 | **.15** | .07 | .07 | .06 | .06 | .05 | .08 | **.71** |  | **.64** | **.61** | **.17** | **.25** | **.26** | **.24** | .04 | -.02 | -.03 | **.10** | **.22** |
| 19. A T3 | **.11** | **.18** | .10 | .08 | **-.13** | **-.23** | **-.23** | **-.27** | .04 | **.14** | **.17** | .12 | .07 | .05 | .09 | .12 | **.61** | **.68** |  | .59 | **.13** | **.21** | **.35** | **.26** | -.02 | -.09 | -.10 | -.05 | **.28** |
| 20. A T4 | .02 | **.15** | .03 | .11 | -.09 | **-.24** | **-.13** | **-.34** | .08 | .11 | **.16** | **.23** | .07 | .05 | .03 | .09 | **.58** | **.64** | **.73** |  | .07 | .10 | .15 | **.31** | .09 | **-.18** | **-.18** | .02 | **.19** |
| 21. C T1 | .07 | .00 | **.14** | .09 | **-.33** | **-.23** | **-.26** | **-.18** | **.28** | **.21** | **.21** | **.14** | **-.11** | **-.16** | **-.13** | **-.23** | **.25** | **.15** | **.17** | **.16** |  | **.70** | **.62** | **.65** | .02 | -.08 | **-.15** | .01 | **-.11** |
| 22. C T2 | .06 | **.14** | **.13** | .08 | **-.22** | **-.35** | **-.18** | **-.25** | **.19** | **.30** | **.17** | **.16** | -.08 | **-.09** | -.09 | **-.16** | **.21** | **.24** | **.23** | **.26** | **.69** |  | **.67** | **.63** | .04 | **-.13** | **-.11** | .04 | -.07 |
| 23. C T3 | .02 | .09 | **.22** | .04 | **-.20** | **-.25** | **-.30** | **-.27** | **.19** | **.23** | **.33** | **.26** | **-.11** | -.10 | -.03 | -.07 | **.19** | **.18** | **.25** | **.28** | **.62** | **.66** |  | **.75** | .04 | **-.15** | **-.14** | .05 | -.08 |
| 24. C T4 | -.01 | .06 | **.21** | **.21** | **-.18** | **-.30** | **-.25** | **-.39** | **.18** | **.16** | **.24** | **.34** | **-.15** | **-.15** | -.05 | -.10 | **.16** | **.18** | **.20** | **.35** | **.60** | **.63** | **.69** |  | .00 | **-.20** | -.14 | .04 | -.15 |
| 25. Education (yrs) | **.10** | **.10** | **.19** | **.15** | -.09 | **-.16** | **-.23** | **-.25** | -.06 | .00 | .08 | .10 | **.33** | **.35** | **.32** | **.32** | .00 | .08 | .04 | .11 | -.06 | -.01 | -.00 | **.12** |  | **-.15** | -.06 | **.56** | **-.27** |
| 26. Subjective health | **-.28** | **-.20** | **-.30** | **-.32** | **.24** | **.22** | **.26** | **.22** | **-.22** | **-.22** | **-.23** | **-.18** | **-.16** | **-.13** | -.10 | **-.14** | **-.19** | **-.19** | **-.20** | **-.16** | **-.09** | **-.16** | **-.18** | **-.15** | **-.21** |  | **.49** | **-.15** | .07 |
| 27. Objective health | **-.12** | **-.10** | **-.30** | **-.26** | **.13** | **.14** | **.18** | **.21** | **-.09** | -.06 | **-.14** | -.09 | **-.10** | -.06 | **-.12** | -.07 | -.05 | -.06 | -.10 | -.04 | -.01 | -.04 | **-.11** | **-.14** | **-.24** | **.45** |  | **-.15** | -.01 |
| 28. Cognitive abilities | **.13** | **.12** | **.19** | **.15** | **-.23** | **-.30** | **-.29** | **-.29** | -.01 | .00 | .05 | .10 | **.31** | **.30** | **.27** | **.27** | .01 | **.10** | .06 | **.12** | -.06 | -.01 | .00 | .05 | **.52** | **-.25** | **-.24** |  | **-.25** |
| 29. Sex | .03 | .05 | -.05 | -.02 | **.29** | **.26** | **.27** | **.23** | .00 | .08 | -.03 | .01 | -.04 | -.02 | .00 | .00 | **.18** | **.14** | .10 | .08 | -.03 | .02 | .01 | -.05 | -.06 | .03 | -.02 | **-.25** |  |

*Note.* Values below the diagonal show associations for the midlife cohort (C50), and values above the diagonal represent correlations of the late life cohort (C30). N= Neuroticism; E = Extraversion; A = Agreeableness; O = Openness; C = Conscientiousness. Values in bold print are significant at *p* ≤ .05
